# Biomimetic Synthesis of Gelatin Polypeptide-Assisted Noble-Metal Nanoparticles and Their Interaction Study

**DOI:** 10.1007/s11671-010-9756-1

**Published:** 2010-09-02

**Authors:** Ying Liu, Xiaoheng Liu, Xin Wang

**Affiliations:** 1 Key Laboratory for Soft Chemistry and Functional Materials (Nanjing University of Science and Technology), Ministry of Education, 210094, Nanjing, China; 2 The School of Chemistry and the School of Materials, The University of Manchester, Oxford Road, Manchester, M13 9PL, UK

**Keywords:** Gelatin, Interaction, Gold nanoparticles, Silver nanoparticles, Bimetallic nanoparticles

## Abstract

Herein, the generation of gold, silver, and silver–gold (Ag–Au) bimetallic nanoparticles was carried out in collagen (gelatin) solution. It first showed that the major ingredient in gelatin polypeptide, glutamic acid, acted as reducing agent to biomimetically synthesize noble metal nanoparticles at 80°C. The size of nanoparticles can be controlled not only by the mass ratio of gelatin to gold ion but also by pH of gelatin solution. Interaction between noble-metal nanoparticles and polypeptide has been investigated by TEM, UV–visible, fluorescence spectroscopy, and HNMR. This study testified that the degradation of gelatin protein could not alter the morphology of nanoparticles, but it made nanoparticles aggregated clusters array (opposing three-dimensional α-helix folding structure) into isolated nanoparticles stabilized by gelatin residues. This is a promising merit of gelatin to apply in the synthesis of nanoparticles. Therefore, gelatin protein is an excellent template for biomimetic synthesis of noble metal/bimetallic nanoparticle growth to form nanometer-sized device.

## Introduction

Noble-metal nanocrystals with small uniform size have attracted more attentions for use in binding biomolecules including proteins, enzymes, DNA due to their huge accessible surface area. Great efforts have been devoted to the development of various synthesis methods for preparing monodisperse nanocrystals. For example, Huang et al. [1] reported a general synthesis strategy for metallic nanocrystals in a two-phase liquid–liquid system, which involves a quite fast nucleation stage overlapped with the growth stage within 10 min. Ideal nanometer-sized devices will combine features of nature's own nanodevices (proteins) such as specific recognition, energy transduction, and cooperativity with the electronic, magnetic, and optical properties of nanomaterials [2-5]. As a result of these, biologic molecules such as proteins and DNA are an ideal type of matrix which acts as biotemplates in the direct chemical synthesis of nanometer-sized devices. For instance, peptides with high affinities and specificities has been used to control the interparticle spacing of aggregated gold colloid through the folding of a helix-loop-helix forming polypeptide on the particle's surface [6]. A number of research groups have investigated the ability of amino acids to act as reducing and stabilizing agent for the synthesis of gold nanoparticles [7-9]. Aspartic acid [7], lysine [10], tyrosine [11], and tryptophan [12,13] have been found to initiate and control the synthesis of gold nanostructures at room temperature. Mandal and colleagues [11,13] have found that tyrosine and tryptophan residues reduced gold and silver ions in solution, resulting in the formation of nanoparticles at room temperature. While peptides lacking tyrosine residues were used to modify the shape and size of gold nanoparticles produced with the aid of Sodium Borohydride, NaH, Potassium Borohydride, etc. [14], polypeptides containing a higher number of tandem peptide repeats were found to be more effective in controlling nanoparticle growth. More recently, CuSe Nanosnakes have been fabricated by using bovine serum albumin polypeptides as biotemplates at room temperature [15].

Gelatin is a soluble polypeptide derived from insoluble collagen, the most abundant protein in animal skin and bone, and has been extensively used for food, pharmaceutical, and medical applications [16]. Gelatin is a material that gels and melts reversibly below the normal human body temperature (37°C). Intensive research has been done on α-helix folding of gelatin from random coils to its conformation in the biologically active form. At elevated temperature, gelatin polypeptide chains exist predominantly in the form of flexible, unfold coils in solution and can be partially renatured to the ordered α-helix upon cooling to room temperature. Due to the presence of functional groups including suspended double bond, –NH_2_, –SH and –COOH, gelatin can be further modified with other bio-molecules for different purposes. Therefore, gelatin is an ideal natural protein for biotemplating nanoparticles into ideal engineering nanometer-sized devices. The biologic impacts of nanoparticles are affected by the nature of the adsorbed protein layer or the protein (bimolecular) corona. Nanoparticles possess more surface area which increases non-specific interactions of amino acid side chains of the gelatin protein with the nanoparticle surface. Nevertheless, little attention has been paid to the interaction between gelatin and noble-metal nanoparticles. This limits the wide use of gelatin polypeptide–mediated noble-metal nanoparticles, particularly in biologic applications. Zhang et al. [17] simply reported that biocompatible gold nanoparticles could be successfully prepared in one step by using gelatin polypeptide upon thermal reduction. In this paper, we researched on the generation of gold, silver and gold–silver bimetallic nanoparticles using gelatin as a reducing and stabilizing agent, and the interaction between nanoparticles and gelatin. In addition, the shape control of gold nanoparticles, against NaCl salt-induced and pH-induced aggregation was discussed. The potential use of nanoparticles in biologic applications and the increasing importance of the emerging field of nanotoxicology, which aims to address the safety of engineered nanoparticles, are well known. Therefore, this paper will be an ignited clue for gelatin polypeptide application in engineering nanometer-sized devices.

## Experimental

### Materials and Methods

Gelatin (type B, extracted from bovine skin) was purchased from Arcos Organics. HAuCl_4_ · 4H_2_O (Au% > 48%) and AgNO_3_ were from Shanghai Chemical Reagent Co. (Shanghai, China). All chemicals were used without further purification.

Morphology analyses of samples were carried out on a JEOL TEM operating at 200 kV. X-ray diffraction (XRD) patterns of the samples were recorded on a Bruker D8 using CuKα radiation (*λ* = 1.5418 Å) in the range 10–70°. The film sample was prepared by the metal nanoparticle colloid drying at room temperature under vacuum. The UV–vis absorption spectra were carried out with a Beijing Eraic UV-1201 spectrometer. Fluorolog-1040 (JobinYvoh Horiba) was used for Fluorescence measurements. ^1^HNMR Watergate water suppression spectra of gelatin solution and gelatin-AuNPs colloid were recorded on a Bruker 400 MHz spectrometer in H_2_O.

### Generation of Gold Nanoparticles

Gold nanoparticles (AuNPs) with various diameters were prepared using gelatin as reducing/stabilizing reagent. The aqueous gelatin solution with different solid concentrations (100 mL) was heated to 80°C with vigorous stirring, then 2.0 mL of HAuCl_4_ solution (0.4 wt%) was added rapidly. The mixed solution was stirred continually for 4 h at 80°C, after which a red gelatin-AuNPs solution was obtained.

To obtain the information on AuNPs stabilized by gelatin, the gelatin backbone was removed by hydrolyzing gelatin-AuNPs in 2 M HCl for 24 h under reflux. Then the solution was characterized with UV–vis and TEM.

### Generation of Silver Nanoparticles

Silver nanoparticles (AgNPs) with various diameters were prepared using gelatin as reducing/stabilizing reagent. The aqueous gelatin solution with different solid concentrations (100 mL) was heated to 80°C. A volume of 2.0 mL of the AgNO_3_ solution (0.4 wt%) was added rapidly with vigorous stirring. After silver nitrate was added into gelatin solution, the color of the solution changed from faint yellow to white at once. This is due to the formation of complex ion of gelatin and silver (I) [18]. The reaction mixture is under the dark environment to avoid the light. The mixed solution was stirred vigorously for 8 h at 80°C, at which a gelatin-AgNPs solution was obtained.

### Generation of Non-Spherical Gold Nanoparticles

The aqueous gelatin solution with different solid concentrations (100 mL) was heated to 80°C. The 2 mL silver seeds (Synthesis according to the procedure in "Generation of Silver Nanoparticles") was added into the gelatin solution. After 10 min, 2.0 mL of the HAuCl_4_ solution (0.4 wt%) was added rapidly under vigorous stirring. The mixed solution was stirred vigorously for 4 h at 80°C, after which a purplish red gelatin-AuNPs solution was obtained.

### Generation of Silver–Gold Bimetallic Nanoparticles (Ag–AuNPs)

Silver–Gold nanoparticles (Ag–AuNPs) with various diameters were prepared using gelatin as reducing/stabilizing reagent. The aqueous gelatin solution with different solid concentrations (100 mL) was heated to 80°C. When stirred vigorously, AgNO_3_ solution (0.4 wt%) was added rapidly. The mixed solution was stirred vigorously for 8 h at 80°C. And then, HAuCl_4_ solution (0.4 wt%) was added under vigorous stirring. The mixed solution turned colorless at once. The colorless solution was stirred vigorously for 4 h at 80°C, after which a purple gelatin-Ag–AuNPs solution was obtained.

## Results and Discussion

### Generation of Gelatin-Directed Gold Nanoparticles (AuNPs) and Their Interaction Study

**Scheme 1:**
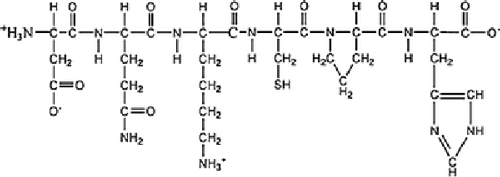
A typical structure of gelatin polypeptide.

**Figure 1:**
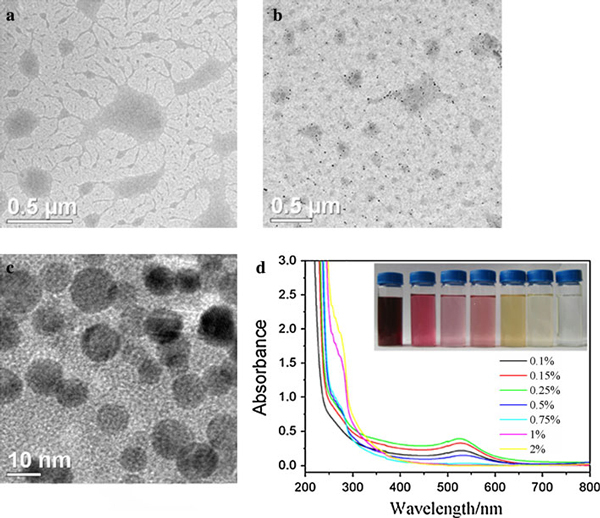
**a TEM image of gelatin; b TEM image of AuNPs (*dark dots*) loaded by gelatin (*gray plates*) synthesized at 80°C (*C*_gelatin_: 0.4%); c The magnified TEM images of b; d UV–vis of the Gelatin-AuNPs synthesized with 0.1, 0.15, 0.25, 0.5, 0.75, 1, and 2% gelatin solutions**. The *inset* is the corresponding optical images of gelatin-AuNPs colloids (the gelatin concentration increasing from *left* to *right*).

In the synthesis of AuNPs, classical reducers such as mercaptoundecanoic acid [19] and macromolecular containing end amino units such as (i.e. PVP-NH_2_) [20] are the most important type of stabilizing and reducing molecule to gold nanoparticles of any size. The functional group of gelatin such as –NH_2_, –SH and –COOH endow it as reducing and stabilizing agent to reduce Au(III) to form gold colloid. As observed in Figure 1, well-dispersed gelatin stabilized gold nanoparticles were prepared according to the synthetic procedure in "Generation of Gold Nanoparticles". Gelatin polypeptide chains with predominately coil conformation are freely soluble in water at elevated temperature (>35°C). However, gelatin molecules linked together to form aggregates when gelatin solution and gelatin-AuNPs colloid were gradually cooled to room temperature (see Figure 1a, b). This is because of protein properties. Proteins are marginally stable because the beneficial interactions that govern the native structure are counterbalanced by a large entropy loss associated with going from a large ensemble of states to a more restricted set of conformations, as well as by the repulsive electrostatic interactions present in the native state [21]. The blank zone in Figure 1a and 1b showed there is some free water in gelatin gel because of the gelatin semi-liquid state at the gelatin concentration of 1% or less [22]. Figure 1b showed that it created a AuNP-loaded gelatin microstructure that all AuNPs embedded in gelatin polypeptide templates instead of gold nanoparticle clusters coated with gelatin or isolated AuNPs clusters departed form aggregated polypeptide. Therefore, AuNPs is not only stabilized by gelatin polypeptides chains but also joined in the self-assembling activities of gelatin polypeptides upon cooling to room temperature. Figure 1d showed the typical absorption band of gold nanoparticles and gelatin was at about 500–550 nm and around 230–300 nm, respectively. When the gelatin concentration is above 0.25%, there is an obvious red shift of the absorption band edge of gelatin residue. This is due to the interaction of gold nanoparticle and gelatin residues.

**Figure 2:**
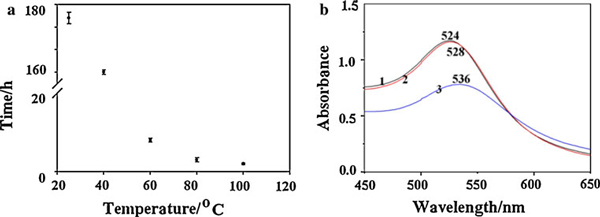
**a Relation between the reaction time and temperature (*C*_gelatin_: 0.4 wt%), The reaction time was determined by UV–vis spectra**. When the typical absorption band of gold nanoparticle was stable, the reaction is considered to be over; **b** UV–vis spectra of gelatin-AuNPs colloids synthesized at different temperature (*1*) 100°C, (*2*) 80°C, (*3*) 60°C.

The amino acid analysis of gelatin is variable, particularly for the minor constituents, depending on raw materials and processes used, proximate values by weight are [23]: glycine 21%, proline 12%, hydroxyproline 12%, glutamic acid 10%, alanine 9%, arginine 8%, aspartic acid 6%, lysine 4%, serine 4%, leucine 3%, valine 2%, phenylalanine 2%, threonine 2%, isoleucine 1%, hydroxylysine 1%, methionine and histidine <1% with tyrosine <0.5% in which methionine [24,25], lysine [10], tyrosine [11] have strong electron-donating properties, which are currently being utilized for the reduction of the Au(III) ion to form Au(0) colloid at room temperature separately. Gold nanoparticles can also be prepared by using glutamic acid upon thermal reduction method [26]. As the ingredient of gelatin polypeptide, methionine [24,25], lysine [10], tyrosine [11] is very little, it took a week for gelatin to reduce Au(III) ion to form metallic Au at room temperature (see Figure 2a). However, HAuCl_4_ can be easily reduced by gelatin polypeptide at higher temperature (>60°C). This may be due to the major ingredient as reducing agent at higher temperature.

**Figure 3:**
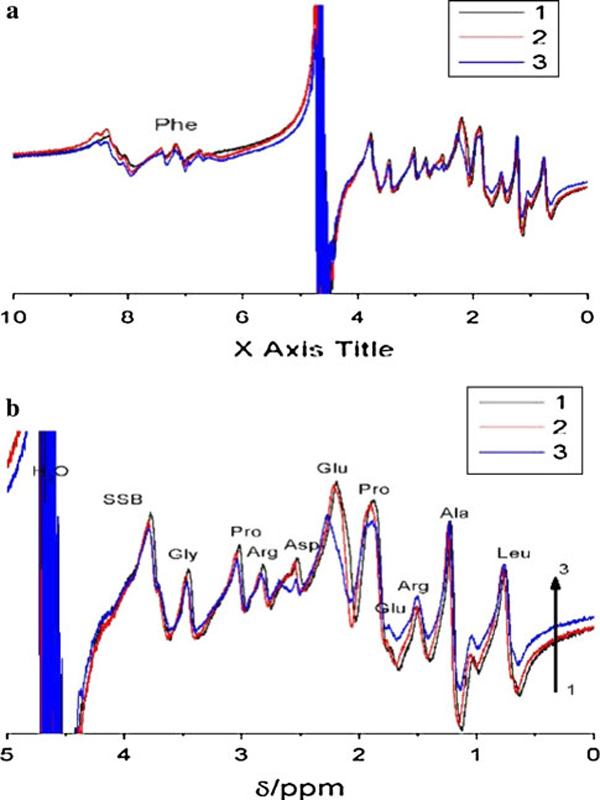
**HNMR spectra of 0.4% gelatin solution and gelatin-Au colloid at room temperature**. (*1*) 0.4% gelatin solution; (*2*) gelatin-Au colloid containing 0.4% gelatin and 0.02 M gold nanoparticles synthesized at 80°C; (*3*) gelatin-Au colloid containing 0.4% gelatin and 0.2 M gold nanoparticles synthesized at 80°C.

Figure 3 showed that there is no obvious line-width change of gelatin residual spectra in a gel state at room temperature. However, the glutamic residue spectra has an obvious change in chemical shift and the intensity before and after gelatin as reducing agent to gold(Ш). This testified that glutamic acid, a natural amino acid with a primary amine opposite carboxylate groups, acted as not only stabilizing agent but also reducing agent to form gold colloid at higher temperature. Figure 3 also showed the interaction between hydrophobic proline residue and gold nanoparticle, although their role in the mineralization process is not as well understood [27].

**Figure 4:**
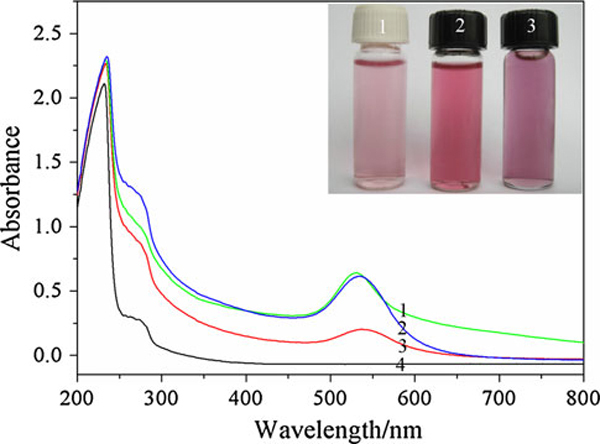
**UV–vis spectra of gelatin-AuNPs synthesized at different pH, respectively (*C*_gelatin_: 0.4 wt%, *T*: 80°C)**. (*1*) pH = 2.4, (*2*) pH = 4.7 (the isoelectric point of gelatin, pI), (*3*) pH = 7.2, (*4*) pure gelatin solution (0.4 wt%). The reaction time was monitored by UV–vis spectra. When the typical absorption band of gold nanoparticle was stable, the reaction is considered to be over. The reaction time of *curve 1*, *2*, *3* was 12, 4, 48 h, respectively. The *inset* is digital images of gelatin-AuNPs corresponding to *curve 1*, *2*, *3*, respectively. The pH of gelatin was adjusted by 0.1 M NaOH and HCl.

This mineralization ability of peptides to gold ion is thought to arise from the amino acid sequence, which contains a tyrosine residue and is rich in serine (hydroxyl-containing amino acid) and hydrophobic amino acids (proline and phenylalanine). The peptide structure, the solution pH, temperature, buffer salt, and electrolyte contents are major factors determining its mineralization ability [27]. As gelatin is polypeptide including praline, glutamic acid, lysine, etc., the reaction parameters such as the solution pH, temperature may affect the gold nanoparticles formation by using gelatin as reducing and stabilizing agent. More recently, Huang et al. [15] have discussed the conformational isomerization effect of BSA polypeptides on the growth of BSA-assisted CuSe nanostructures in different pH condition. Therefore, we synthesized the AuNPs in gelatin solution (0.4 wt%, 80°C) at different pH to study the mineralization ability changing of gelatin to gold(Ш). Figure 4 showed that the typical color of AuNPs changed from light red, wine red to purple-red, and there is a red shift and the intensity changed in the maximum of UV–vis absorption band with the increased pH. Hence, the size of AuNPs can be controlled not only by the mass ratio of gelatin to HAuCl_4_ but also by pH alteration. Nevertheless, the reaction time obviously delayed when the typical absorption band of AuNPs is stable at pH different from the isoelectric point(pI) [pH = 2.4, 12 h; pH = 4.7, 4 h; pH = 7.2, 48 h]. This is mainly because of the amphoteric properties and conformation isomerization of polypeptides as the pH value changes. As gelatin polypeptides contains both acidic and basic functional groups, at a pH below the isoelectric point (pI, 4.7), gelatin carries a net positive charge, i.e. the amino has partly changed into –NH_3_^+^. Above pI of 4.7, gelatin carries a net negative charge, i.e. the carboxyl unit has changed into –COO^-^, and sulfhydryl group has changed into –S^-^. The electron-donating ability of gelatin would be greatly changed. The reducing ability of gelatin residues to Au(III) would be greatly decreased by adjusting pH. Furthermore, we discovered that gelatin could not be used as reducing agent for AuNPs at pH > 9 or pH < 1. This is attributed to that gelatin structure in solution decides its function to reduce gold (Ш).

$$\begin{array}{c} {}^{+}{\text{H}}_{3}\text{N--gelatin--COOH}{\overset{\text{pH}<\text{pI}}{\leftarrow }}^{+}{\text{H}}_{3}{\text{N--gelatin--COO}}^{-}\\ \to \text{pH}>{\text{pIH}}_{2}{\text{N--gelatin--COO}}^{-} \end{array}$$

**Figure 5:**
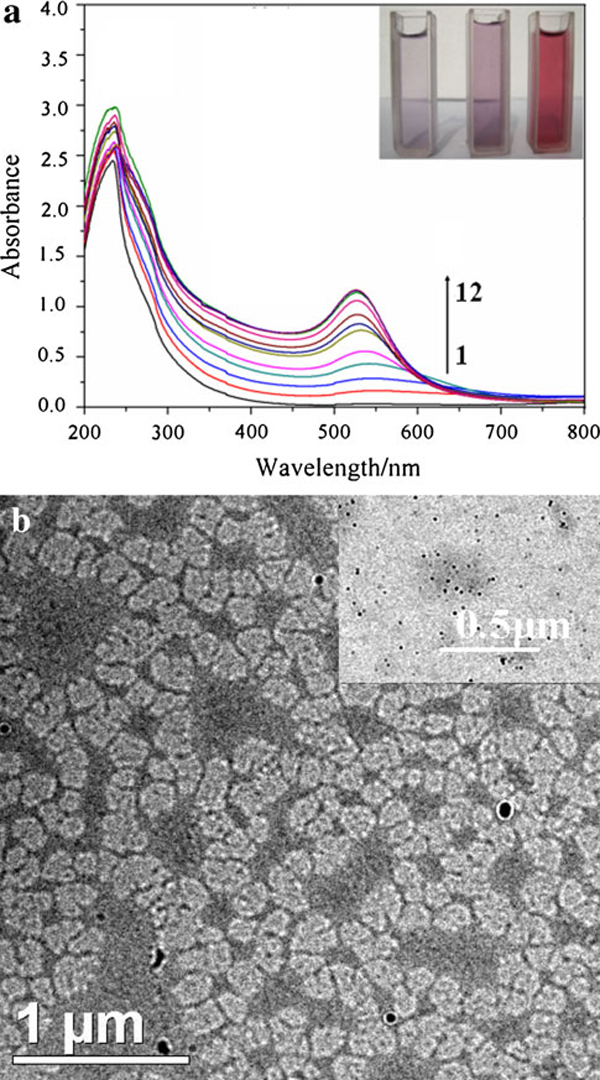
**a Time evolution of the UV–vis spectra of gelatin-AuNPs synthesized at 80°C (*C*_gelatin_: 0.4 wt%)**. The time intervals are (*1*) 10 min, (*2*) 20 min, (*3*) 40 min, (*4*) 60 min, (*5*) 80 min, (*6*) 100 min, (*7*) 120 min, (*8*) 140 min, (*9*) 160 min, (*10*) 180 min, (*11*) 240 min, (*12*) 360 min. The *inset* in **a** is digital images of gelatin-AuNPs (from *left* to *right*) corresponding to the *curve 2*, *3*, *10*, respectively; **b** TEM images of gelatin-AuNPs (*curve 3*), the *inset* in **b** is the TEM image of gelatin-AuNPs corresponding to the *curve 10*.

The interaction between the AuNPs and gelatin polypeptide chains at different stages was monitored by UV–vis absorption spectroscopy and TEM. As shown in Figure 5a, the intensity of the surface plasmon absorption increased with the increase in reaction time, which indicated the continued reduction of the metal ions with gelatin. The inset in Figure 5a showed the color of solution changed form light purple, purple into wine red, which indicated the nanoparticle morphology changing at different reaction stage. In addition, an obvious 25-nm blue shift from the surface plasmon absorption band of AuNPs with increased reaction time was observed. Figure 5b showed the initial stage of the reaction (Curve 3, 40 min), size of AuNPs with irregular shape at about 50–80 nm. The UV–vis absorption peak is about 550 nm. After this stage, complexes of gelatin-modified AuNPs changed into smaller particles stabilized by gelatin backbone (see the inset in Figure 5b). It maybe due to thermal dissociation of complexes of gelatin-modified AuNPs [15]. But the initial growth of nanoparticles by using gelatin polypeptide as reducing and stabilizing agent is not clear. The more rational scheme may be acquired by in situ SAXS and XANES using synchrotron radiation [28].

**Figure 6:**
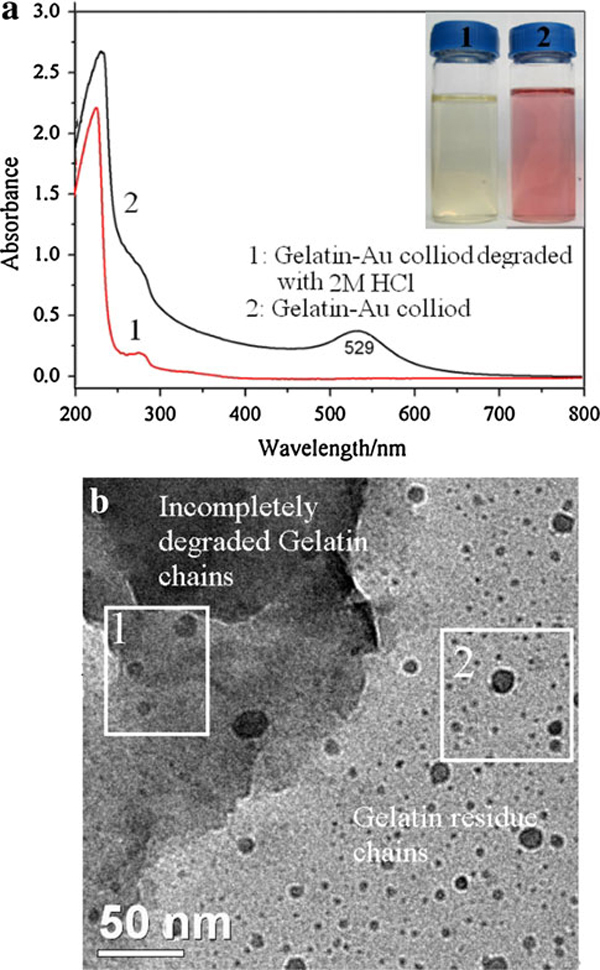
**a UV–vis spectra of gelatin-AuNPs and gelatin-AuNPs degraded by 2 M chlorhydric acid (*C*_gelatin_: 0.4 wt%)**. The *inset* in **a** is the digital image of *Curve 1* (*left*) and *2* (*right*) colloids; **b** TEM image of incompletely degraded gelatin-AuNPs colloid.

**Figure 7:**
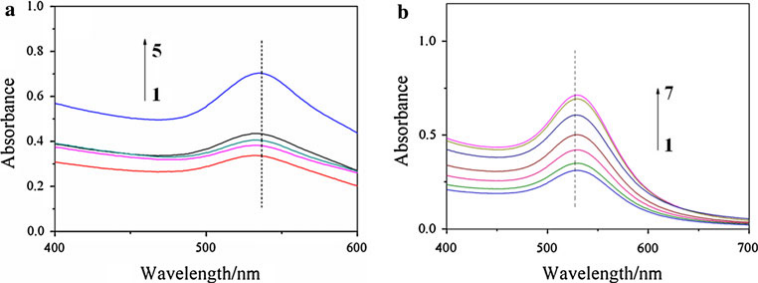
**UV–vis spectra of AuNPs colloid against pH (**a**) and NaCl (**b**)**. The pH in **a** are (*1*) 0.2, (*2*) 2.0, (*3*) 4.7 (pI), (*4*) 9.0, (*5*) 13.0. The pH of gelatin-AuNPs colloid was adjusted by 0.01 M NaOH and HCl solution; The concentrations of NaCl in **b** are (*1*) 0.9%, (*2*) 2%, (*3*) 4%, (*4*) 8%, (*5*) 10%, (*6*) 12%, (*7*) 36.5%.

**Figure 8:**
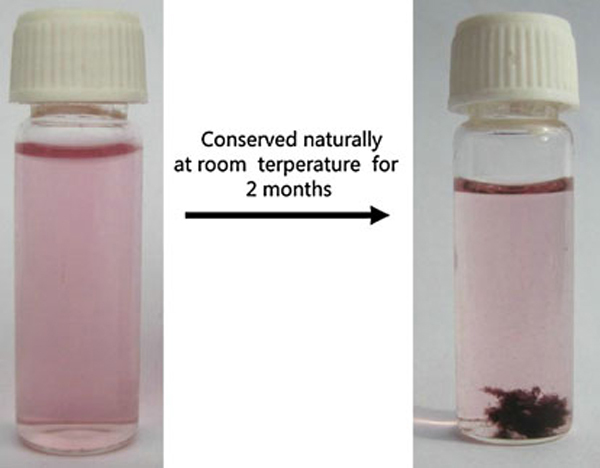
**The digital image of gelatin-AuNPS colloids (*left*) and gelatin-AuNPS colloids containing fungoids (*right*)**.

Gelatin is prone to degradation on incubation at elevated temperature in the absence of proteases. The incubation parameters such as temperature, time, pH, and salt ions have great effect on gelatin degradation [29]. Therefore, the gelatin degradation has been partly carried out in the process of gold nanoparticle growth at higher temperature. To obtain the information on AuNPs stabilized by gelatin, the gelatin backbone was removed by hydrolyzing gelatin-AuNPs in 2 M HCl. Figure 6a showed that the plasmon resonance band of AuNPs at 529 nm has disappeared. But the size and shape of AuNPs stabilized by gelatin residues (marked by pane 2 in Figure 6b) has no obvious difference with AuNPs stabilized by gelatin as well as incompletely degraded gelatin by comparing with Figures 1 and 6b. The plasmon resonance band of AuNPs depends on the morphology of nanoparticles and the dielectric properties of the surrounding medium [30]. Exploring the reason of this alteration on the plasmon resonance of AuNPs, we studied the stability of AuNPs against pH and salt (NaCl) at room temperature (see Figure 7). There, besides the decrease in plasmon resonance peak intensity of AuNPs induced by diluting the gelatin-AuNPs colloid with NaOH, HCl or NaCl solution, Figure 7 showed that the plasmon resonance peak of AuNPs remained stable by altering pH and NaCl concentration of the gelatin-AuNPs colloid, even at lower pH and higher NaCl concentration. When the three-dimensional α-helix folding structure of gelatin was destroyed by the degradation of gelatin with the acid, gelatin polypeptides changed into gelatin residues. Therefore, Curve 1 in Figure 6a showed the plasmon resonance band of AuNPs aggregates (including many separated AuNPs) stabilized by gelatin three-dimensional α-helix folding chains and Curve 2 showed the plasmon resonance band of separated AuNPs stabilized by gelatin three-dimensional α-helix folding chains. Furthermore, the gelatin solution degraded slowly and is suitable for fungal growth at room temperature. The gelatin template has changed into fungal templates for gold nanoparticles (see Figure 8) (the fungal species is very complicated because it originated from fungoid in air) [31].

**Figure 9:**
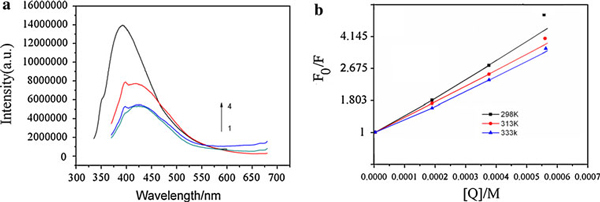
**a Fluorescence emission gelatin-AuNPs solutions (*1*–*3*, Excitation at 350 nm) and gelatin (*4*, Excitation at 335 nm) at the concentration of gelatin is 0.4 wt%, the concentrations of AuNPs are (1) 0.0006 M, (2) 0.0004 M, (3) 0.0002 M; b Stern–Volmer plot for gelatin-AuNPs**.

The interaction between gelatin and gold nanoparticles has been investigated by Fluorescence techniques under pI. The potential interaction between gelatin and AuNPs is implied by the intrinsic fluorescence peaks at 395 nm that mainly originated from the lysine, tryptophan, and tyrosine residues. Figure 9a showed gold nanoparticles dramatically quenching the intrinsic fluorescence of gelatin protein. The fluorescence quenching mechanism can be analyzed quantitatively at 298 K with the Stern–Volmer equation [32].

$$\frac{F_{0}}{F}=1+K_{\text{SV}}[Q]$$

where *F*_0_ and *F* are the relative fluorescence intensities of gelatin at 395 nm in the absence and presence of quencher, AuNPs, respectively; *K*_SV_ is the Stern–Volmer quenching constant, and [*Q*] is the concentration of quencher (AuNPs). The Stern–Volmer plots at 298, 313, and 333 K showed that the fluorescence quenching at 298 nm by AuNPs is approximately in agreement with the Stern–Volmer equation (Figure 9b) at lower concentration. The quenching constant decreases with increasing temperature, which indicates that the quenching mechanism mainly arises from static quenching [33]. As such, a ground state complex is probably formed between gelatin and AuNPs that leads to this intrinsic fluorescence quenching. For AuNPs where the particle core is surrounded by a narrower band gap shell, one can rather expect luminescence quenching due to the possibility of the photo-generated charge-carrier recombination within the narrower gap shell than an increase of luminescence efficiency.

### Generation of Gelatin-Silver Nanoparticle (AgNPs), Non-Spherical AuNPs and Ag–Au Bimetallic Nanoparticles

**Figure 10:**
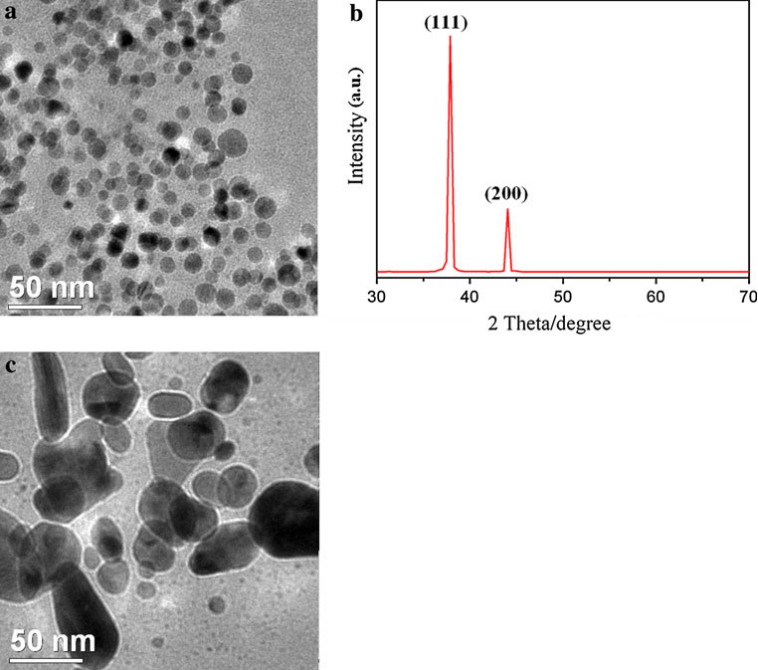
**a TEM image of gelatin-AgNPs synthesized at 90°C (*C*_gelatin_: 0.4 wt%, $V_{{\text{AgNO}}_{3}}$: 2 mL); b XRD of gelatin-AgNPs power obtained from the sample in a; c TEM image of gelatin-AgNPs synthesized at 90°C (*C*_gelatin_: 0.4 wt%, $V_{{\text{AgNO}}_{3}}$: 4 mL). AgNO_3_ was added in gelatin solution in two times**. At first, 0.2 mL AgNO_3_ was added. The other AgNO_3_ was added into reaction mixture after 10 h.

Figure 10a showed that spherical AgNPs could be prepared by using gelatin polypeptide as reducing and stabilizing agent. The large face on the gelatin-directed silver crystals is (111), verified by XRD (see Figure 10b). The (111) family of faces is those with the fewest number of broken bonds per atom and the lowest surface energy. That is, bringing in another silver atom from the gelatin solution above the silver–water interface to the growing crystal is least energetically favored at the (111) faces. Most face-centered cubic (fcc) metals, including gold, silver have cuboctahedral equilibrium shape with (111) facets that have a slightly lower surface energy than the (100) facets, reflected as (111) facets having a slightly larger area than (100) facets. At present, it is widely accepted that controlling the shape of metal nanoparticles in liquid media requires the use of an appropriate seed or soft structure–directing agent such as surfactant, stabilizer, and foreign metal ion [34]. Therefore, we synthesized non-spherical AgNPs and AuNPs by controlling the crystal facet growth. Figure 10c showed non-spherical AgNPs was formed by adding AgNO_3_ into gelatin solution in two times. The AgNPs formed in the first step was used as silver seeds.

**Figure 11:**
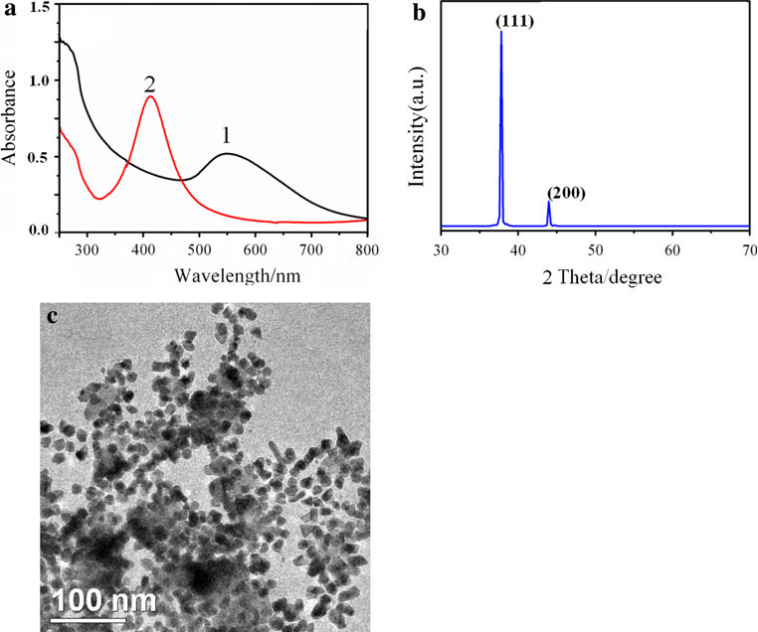
**a UV–vis spectra of gelatin-AuNPs (*1*) synthesized at 80°C ($V_{{\text{HAuCl}}_{4}}$: 4 mL) and AgNPs precursor (*2*); b XRD of gelatin-AuNPs film dried at room temperature under vacuum**. **c** TEM image of gelatin-AuNPs.

The synthesis of non-spherical AuNPs was also prepared using gelatin-directed AgNPs as crystal seeds. Figure 11a showed that the plasmon resonance band of AgNPs at 413 nm in gelatin-AuNPs colloid disappeared. There was a broad plasmon resonance band of AuNPs at 559 nm. The large face on the gelatin-directed gold crystals is also (111) (see Figure 11b). Figure 11c showed the formation of triangle and rod AuNPs. The shape of the formed nanoparticles in this article would be discussed at length in a later paper.

**Figure 12:**
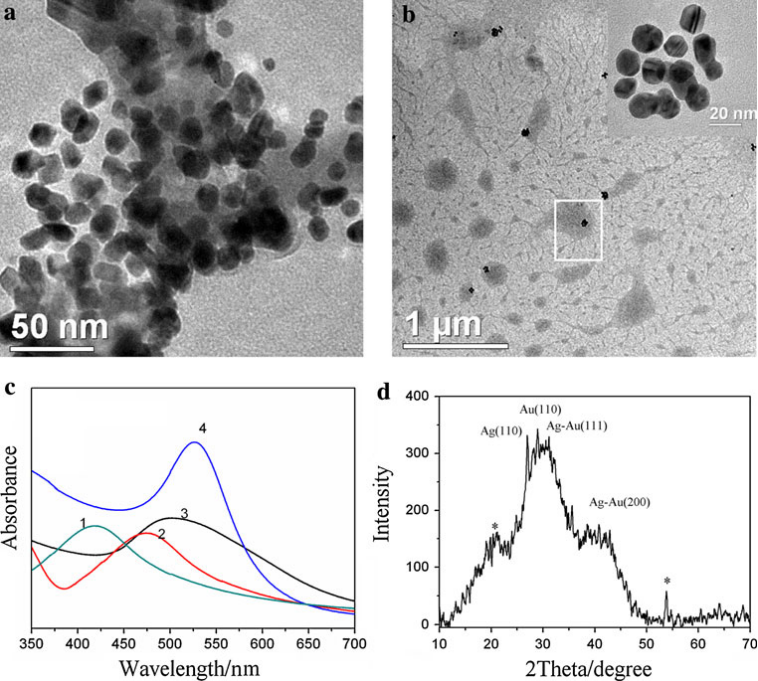
**a, b TEM images of gelatin-Ag–AuNPs with different ratios of silver to gold synthesized at 80°C**. **a ***n*_Ag_/*n*_Au_ = 2.4, $V_{{\text{AgNO}}_{3}}$: 2 mL, $V_{{\text{HAuCl}}_{4}}$: 1 mL; **b ***n*_Ag_/*n*_Ag_ = 1.2, $V_{{\text{AgNO}}_{3}}$: 2 mL, $V_{{\text{HAuCl}}_{4}}$: 2 mL, the *inset* in **b** is magnification of the image marked by *pane* in **b**; **c** UV–vis spectra of Ag–AuNPs, AgNPs and AuNPs. (*1*) AgNPs (*C*_gelatin_: 0.4 wt%, $V_{{\text{AgNO}}_{3}}$: 2 mL), (*2*) Ag–AuNPs in Figure 12a, (*3*) Ag–AuNPs in Figure 12b, (*4*) AuNPs (*C*_gelatin_: 0.4 wt%, $V_{{\text{HAuCl}}_{4}}$: 2 mL); **d** XRD of gelatin-Ag–AuNPs film dried at room temperature under vacuum. *Peaks* marked with *stars* arise from gelatin organic phase.

Bimetallic nanoparticles have recently attracted great research interests due to their potential applications in technologies such as catalysis, electronics and optical devices. The chemical and physical properties of bimetallic particles can be tuned not only by varying their size but also their composition. We synthesized Ag–Au bimetallic nanoparticles by using gelatin co-reduction action due to the fact that gelatin can be used as a reducing and stabilizing agent for gold and silver ion. Figure 12 showed that Au–Ag alloy clusters including isolated nanoparticles with different dimensions were embedded in gelatin polypeptide templates. Figure 12c showed Ag–Au clusters synthesized by using gelatin as a stabilizing and reducing agent are intermixed structures [35]. As the plasmon frequency of intermixed bimetallic clusters varies smoothly with composition between that of the pure Ag and pure Au clusters, the plasmon absorbance band of Ag–Au nanoparticles (Curve 2, 3 in Figure 12c) has been shifted to correspond with pure AgNPs (Curve 1) and AuNPs (Curve 4). Furthermore, Figure 12d showed the XRD pattern obtained for the Au–Ag nanoparticles-modified gelatin. Here, four different characteristic peaks obtained were Ag (110), Au (110), Au–Ag (111), Au–Ag (200). All these four XRD peaks clearly validate the presence of Au–Ag bimetallic nanoparticles [36]. And the XRD intensity of Au–Ag alloy nanoparticles decreased greatly compared to the single metal nanoparticles. This difference is mainly because atoms in Au–Ag alloy have insignificant deviations from an ideal fcc lattice [37].

## Conclusions

In this paper, we reported the generation of Au, Ag and Ag–Au bimetallic nanoparticles by using gelatin as reducing and stabilizing agent, the interaction between nanoparticles and gelatin polypeptides, and shape control of gold nanoparticles, against salt-induced (NaCl) and pH-induced aggregation. This study reveals that metal nanoparticles are not only stabilized by gelatin polypeptides chains but also joined in the self-assembling activities of gelatin polypeptides upon cooling to room temperature. It first showed that the major ingredient in gelatin polypeptide, glutamic acid, acted as reducing agent to biomimetically synthesize noble metal nanoparticles at 80°C. The destruction of the three-dimensional α-helix folding structure by gelatin polypeptide acid degradation could not alter the morphology of nanoparticles, but it made nanoparticles aggregated clusters array (opposing three-dimensional α-helix folding structure) into isolated nanoparticles stabilized by gelatin residues. This is a promising merit of gelatin to apply in the synthesis of nanoparticles. Therefore, gelatin protein is an excellent template to regulate the nanoparticle growth. Gelatin-noble-metal colloids can be also used as an excellent engineering nanometer-sized device in bio-detection, optical devices and so on.
